# Dietary intake of vitamin C and gastric cancer: a pooled analysis within the Stomach cancer Pooling (StoP) Project

**DOI:** 10.1007/s10120-024-01476-8

**Published:** 2024-03-04

**Authors:** Michele Sassano, Monireh Sadat Seyyedsalehi, Giulia Collatuzzo, Claudio Pelucchi, Rossella Bonzi, Monica Ferraroni, Domenico Palli, Guo-Pei Yu, Zuo-Feng Zhang, Lizbeth López-Carrillo, Nuno Lunet, Samantha Morais, David Zaridze, Dmitry Maximovich, Vicente Martín, Gemma Castano-Vinyals, Jesús Vioque, Sandra González-Palacios, Mary H. Ward, Reza Malekzadeh, Mohammadreza Pakseresht, Raul Ulises Hernández-Ramirez, Malaquias López-Cervantes, Eva Negri, Federica Turati, Charles S. Rabkin, Shoichiro Tsugane, Akihisa Hidaka, Areti Lagiou, Pagona Lagiou, M. Constanza Camargo, Maria Paula Curado, Stefania Boccia, Carlo La Vecchia, Paolo Boffetta

**Affiliations:** 1 Department of Medical and Surgical Sciences, University of Bologna, Via Massarenti 9, 40138 Bologna, Italy; 2 Department of Clinical Sciences and Community Health, University of Milan, Milan, Italy; 3 Cancer Risk Factors and Life-Style Epidemiology Unit, Institute for Cancer Research, Prevention and Clinical Network, ISPRO, Florence, Italy; 4 Medical Informatics Center, Peking University, Beijing, China; 5 Department of Epidemiology, UCLA Fielding School of Public Health and Jonsson Comprehensive Cancer Center, Los Angeles, CA USA; 6 Mexico National Institute of Public Health, Cuernavaca, Mexico; 7 EPIUnit, Instituto de Saúde Pública da Universidade Do Porto, Porto, Portugal; 8 Laboratório Para a Investigação Integrativa E Translacional Em Saúde Populacional (ITR), Porto, Portugal; 9 Departamento de Ciências da Saúde Pública E Forenses E Educação Médica, Faculdade de Medicina, Universidade Do Porto, Porto, Portugal; 10 Department of Clinical Epidemiology, N.N. Blokhin National Medical Research Center for Oncology, Moscow, Russia; 11 Consortium for Biomedical Research in Epidemiology and Public Health (CIBERESP), Madrid, Spain; 12 Instituto de Biomedicina, Universidad de León, León, Spain; 13 Barcelona Institute for Global Health-ISGlobal, Barcelona, Spain; 14 IMIM (Hospital del Mar Medical Research Institute), Barcelona, Spain; 15 Universitat Pompeu Fabra (UPF), Barcelona, Spain; 16 Instituto de Investigación Sanitaria y Biomédica de Alicante, Universidad Miguel Hernandez (ISABIAL-UMH), Alicante, Spain; 17 Division of Cancer Epidemiology and Genetics, National Cancer Institute, National Institutes of Health, Rockville, MD USA; 18 Digestive Oncology Research Center, Digestive Disease Research Institute, Tehran University of Medical Sciences, Tehran, Iran; 19 Department of Agricultural, Food and Nutritional Sciences, University of Alberta, Edmonton, Canada; 20 Nutritional Epidemiology Group, Centre for Epidemiology and Biostatistics, University of Leeds, Leeds, UK; 21 Department of Biostatistics, Yale School of Public Health, New Haven, CT USA; 22 Facultad de Medicina, National Autonomous University of Mexico (UNAM), Coyoacán, Mexico; 23 Division of Cohort Research, National Cancer Center Institute for Cancer Control, Tokyo, Japan; 24 International University of Health and Welfare Graduate School of Public Health, Tokyo, Japan; 25 Division of Epidemiology, National Cancer Center Institute for Cancer Control, Tokyo, Japan; 26 Department of Diabetes and Endocrinology, JCHO Tokyo Yamate Medical Centre, Tokyo, Japan; 27 Department of Public and Community Health, School of Public Health, University of West Attica, Athens, Greece; 28 Department of Hygiene, Epidemiology and Medical Statistics, School of Medicine, National and Kapodistrian University of Athens, Athens, Greece; 29 Department of Epidemiology, Harvard T.H. Chan School of Public Health, Boston, MA USA; 30 Centro Internacional de Pesquisa, A. C. Camargo Cancer Center, São Paulo, Brasil; 31 Section of Hygiene, University Department of Life Sciences and Public Health, Università Cattolica del Sacro Cuore, Rome, Italia; 32 Department of Woman and Child Health and Public Health, Fondazione Policlinico Universitario A. Gemelli IRCCS, Rome, Italy; 33 Stony Brook Cancer Center, Stony Brooke University, Stony Brook, NY USA; 34 Department of Family, Population and Preventive Medicine, Renaissance School of Medicine, Stony Brook University, Stony Brook, NY USA

**Keywords:** Gastric cancer, Vitamin C, Diet, Pooled analysis, Consortium, Case–control

## Abstract

**Background:**

Previous studies suggest that dietary vitamin C is inversely associated with gastric cancer (GC), but most of them did not consider intake of fruit and vegetables. Thus, we aimed to evaluate this association within the Stomach cancer Pooling (StoP) Project, a consortium of epidemiological studies on GC.

**Methods:**

Fourteen case–control studies were included in the analysis (5362 cases, 11,497 controls). We estimated odds ratios (ORs) and corresponding 95% confidence intervals (CIs) for the association between dietary intake of vitamin C and GC, adjusted for relevant confounders and for intake of fruit and vegetables. The dose–response relationship was evaluated using mixed-effects logistic models with second-order fractional polynomials.

**Results:**

Individuals in the highest quartile of dietary vitamin C intake had reduced odds of GC compared with those in the lowest quartile (OR: 0.64; 95% CI: 0.58, 0.72). Additional adjustment for fruit and vegetables intake led to an OR of 0.85 (95% CI: 0.73, 0.98). A significant inverse association was observed for noncardia GC, as well as for both intestinal and diffuse types of the disease. The results of the dose–response analysis showed decreasing ORs of GC up to 150–200 mg/day of vitamin C (OR: 0.54; 95% CI: 0.41, 0.71), whereas ORs for higher intakes were close to 1.0.

**Conclusions:**

The findings of our pooled study suggest that vitamin C is inversely associated with GC, with a potentially beneficial effect also for intakes above the currently recommended daily intake (90 mg for men and 75 mg for women).

**Supplementary Information:**

The online version contains supplementary material available at 10.1007/s10120-024-01476-8.

## Introduction

Gastric cancer (GC) is currently the fifth most common cancer type and the fourth leading cause of cancer death worldwide, with an estimated 1.1 million new cases and 770,000 deaths in 2020 [1]. Although incidence rates have been declining over recent decades [1, 2], an increase in incidence among adults younger than 50 years has been reported in several populations [3], and it has been suggested that the overall burden of GC will increase in the future, reaching around 1.8 million new cases and 1.3 million deaths per year by 2040 [4].

Among the factors which may influence GC risk, diet could play a relevant role [5, 6]. A recent umbrella review evaluating the effect of 147 unique diet-related exposures on GC risk did not find strong evidence for any of the investigated associations [7]. When considering evidence from prospective primary studies only, suggestive positive associations were reported for heavy alcohol consumption, salted fish consumption, and waist circumference, while a suggestive inverse association was observed for a healthy lifestyle score [7]. Additionally, intake of fruit and vegetables might influence GC risk, likely through their content in antioxidants including some vitamins [7]. Among them is vitamin C (ascorbic acid), which is a water-soluble vitamin that can be found in citrus and other fruits and vegetables. Vitamin C is an essential nutrient in humans and is involved in the regulation of a number of biological mechanisms, including iron metabolism, collagen and carnitine synthesis, catabolism of tyrosine and demethylation of proteins, DNA and RNA, and it also exerts both antioxidant and pro-oxidant activity at micromolar and millimolar concentrations, respectively [8]. Vitamin C might have antineoplastic effects in the stomach by reducing oxidative stress and subsequent cellular and DNA damage [9], inhibiting the formation of N-nitroso compounds and other carcinogens [10, 11], and potentially interfering with *Helicobacter pylori* infection, which is recognized as the main risk factor for GC [6, 12].

Pooled estimates from recent meta-analyses of observational studies seem to confirm an inverse association between dietary vitamin C and GC risk [13–15]. However, most of the studies included in these meta-analyses did not adjust for participants’ intake of fruit and vegetables [13–15], which could thus explain observed associations through the effect of other nutrients and antioxidants, considered either individually or jointly. Also, the current evidence on dietary vitamin C and GC was considered too limited by the World Cancer Research Fund to draw a conclusion in its 2018 report [11]. Together with the lack of adjustment for intake of fruit and vegetables in previous epidemiological studies [13–15], the number of those conducted so far reporting detailed results according to GC subsites and, especially, histological types, is limited [13, 14]. Furthermore, potential interactions between intake of vitamin C and risk factors for GC, such as tobacco smoking, alcohol drinking, and *H. pylori* infection, have not been thoroughly investigated [16].

We evaluated the association between dietary intake of vitamin C and GC accounting also for the potential confounding effect of fruit and vegetable consumption, as well as the occurrence of potential interactions between vitamin C and other factors, within the Stomach cancer Pooling (StoP) Project, an international consortium of epidemiological studies on GC [17].

## Materials and methods

For the present analysis, version 3.2 of the StoP dataset was used, which includes 34 case–control or nested case–control studies totaling 13,121 GC cases and 31,420 controls. The StoP Project was established in 2012 as an international collaborative effort with the aim to elucidate determinants of risk and outcome of GC [17]. Data harmonization of core variables, such as those related to sociodemographic and some lifestyle factors, was carried out at the Coordinating Center in Milan, Italy after research teams shared the data from their previously conducted case–control or nested case–control studies.

Fourteen studies participating in the StoP Project had available information on dietary intake of vitamin C and total energy intake and were thus included in this analysis (5362 cases, 11,497 controls [the flowchart describing the inclusion of study participants is reported in Supplementary Fig. 1]). Among them, seven were from Europe (two from Italy [18, 19], two from Spain [20, 21], and one each from Greece [22], Russia [23], and Portugal [24]), three from Asia (one each from China [25], Japan [26], and Iran [27]), and four from America (one from the USA [28] and three from Mexico [29–31]). Of the studies included in the analysis, eight were community-based [19, 20, 24, 25, 27–30] and six were clinic-based [18, 21–23, 26, 31] (Supplementary Table 1).

The participating studies were conducted in accordance with applicable laws, regulations and guidelines for the protection of human subjects. Furthermore, the StoP Project was approved by the University of Milan Review Board (reference 19/15, April 1, 2015).

### Outcome and exposure assessment

In this analysis, cases were individuals with histologically confirmed GC. Information about histological type (intestinal, diffuse, other, the latter including mixed, undifferentiated, and unclassified type) and subsite (cardia, noncardia) of GC was available for ten [18–21, 23, 24, 27–29, 31] and eleven [18–24, 26–29] studies, respectively.

Within each study, participants’ intake of vitamin C (mg/day) was computed from food-frequency questionnaires (FFQs) using country-specific food composition tables. Subsequently, based on the study-specific distribution of intake among controls, dietary vitamin C was categorized into quartiles. Intake of vitamin C from supplements was excluded from the analysis since information on supplements was available only for four studies (Russia [23], China [25], Portugal [24], and Japan [26]) among those with available data on dietary vitamin C intake, with variable degree of detail on the specific type of supplement taken and with very limited data on the dose.

In each study, structured questionnaires were used to collect information on participants’ sociodemographic and lifestyle characteristics.

### Statistical analysis

We estimated odds ratios (ORs) and corresponding 95% confidence intervals (CIs) for the association between dietary intake of vitamin C and GC using mixed-effects logistic models, with a random intercept for each study. Model 1 was adjusted for sex (male, female), age (≤ 40 years, 41–50 years, 51–60 years, 61–70 years,  > 70 years), socioeconomic status (low, intermediate, or high, according to study-specific definitions based on education, income, or occupation), tobacco smoking status (never, former, current), alcohol drinking status (never, ever), and total energy intake (continuous). Model 2 was adjusted for the same set of covariates as model 1 and, additionally, for body mass index (BMI: underweight [< 18.5 kg/m^2^], normal weight [18.5–24.9 kg/m^2^], overweight [25–29.9 kg/m^2^], obese [≥ 30 kg/m^2^]) and intake of fruit and vegetables (low, intermediate, high, according to study-specific tertiles). Model 3 included the same covariates as model 2 and individuals in the highest and lowest 1% of vitamin C intake were excluded from the analysis. Model 4 was the same as model 2, except for total energy intake being modelled using a second-order fractional polynomial [32]. For all models, missing values of covariates were coded as a separate category for categorical variables, or replaced with the study-specific median values among controls together with dummy variables indicating replacements for continuous variables. Linear trends were assessed in similar models with intake of vitamin C in quartiles considered as a continuous variable. Additionally, for both the point estimate of the OR and the limit of its 95% CI closer to unity, we computed the E-value, which represents the minimum strength of association that an unmeasured confounder should have with both the exposure and the outcome to fully explain away the observed association between the exposure and the outcome, conditional on measured confounders [33].

Moreover, we stratified results according to the following: subsite (cardia, noncardia), histological type (intestinal, diffuse, other), type of study (clinic-based, community-based), sex (male, female), age (≤ 60 years,  > 60 years), socioeconomic status (low, intermediate, high), BMI (underweight, normal weight, overweight, obese), smoking status (never, former, current), drinking status (never, ever), *H. pylori* seropositivity (negative, positive), and intake of fruit and vegetables (low, intermediate, high, according to study-specific tertiles). Data about *H. pylori* seropositivity were available for seven studies, and in four of them, it was assessed through enzyme-linked immunosorbent assay (ELISA) [23, 26, 29, 31], in one using multiplex serology [20], and in two with both ELISA and Western blot testing [24, 27]. Furthermore, we performed a sensitivity analysis by comparing all cases to seropositive controls, assuming that *H. pylori* infection be a necessary cause of GC, regardless of results of serological tests [17, 34]. Stratified analyses were adjusted for sex, age, socioeconomic status, tobacco smoking status, alcohol drinking status, BMI, intake of fruit and vegetables, and total energy intake. We tested for heterogeneity across strata using likelihood ratio tests comparing the models including the interaction terms between quartiles of vitamin C intake and the stratification variable with those excluding them.

Furthermore, we evaluated potential additive interactions between dietary vitamin C (higher vs. lower than the study-specific median intake among controls) and the following variables: age (≤ 60 years,  > 60 years), sex (female, male), socioeconomic status (high, low/intermediate), tobacco smoking status (non-current, current), alcohol drinking status (never, ever), BMI (overweight/obese, normal weight), *H. pylori* seropositivity (negative, positive), intake of fruit and vegetables (high, intermediate/low), and intake of salt (low, intermediate/high). To this aim, we estimated the relative excess risk due to interaction (RERI) and the attributable proportion (AP) due to interaction and computed their 95% CI using the delta method [35, 36], after recoding variables of interest to use the stratum with the lowest OR of GC when both factors were considered jointly as the reference category [37].

Additionally, we assessed the dose–response relationship between dietary intake of vitamin C and GC with similar logistic mixed-effects models. We assessed its linearity by treating dietary vitamin C as a continuous variable in the model, and nonlinearity with first- and second-order fractional polynomials. We ran two different models adjusted for the same covariates as model 1 and model 2 described above for the main analysis, and in both cases we excluded from the analysis individuals in the lowest and in the highest 1% of intake of vitamin C. The model considered as the best fitting was the one with the lowest deviance [32].

All analyses were carried out using Stata software version 17 (StataCorp LLC. College Station. TX).

## Results

Study participants’ characteristics are reported in Table 1 and Supplementary Table 2. More cases were male (61.88% vs. 54.64%), older than 60 years (63.37% vs. 57.87%), and had low socioeconomic status (57.29% vs. 45.66%) compared with controls. In addition, a higher proportion of cases than controls were obese (21.34% vs. 19.06%), current smokers (26.58% vs. 24.14%), and ever drinkers (64.85 vs. 63.29%). Similarly, *H. pylori* seropositivity (63.82% vs. 61.86%) was more common among cases than among controls when considering only participants from the seven studies with available information [20, 23, 24, 26, 27, 29, 31]. A larger proportion of controls reported high intake of fruit and vegetables compared with cases. Most cases were of noncardia (57.03%) and intestinal type GC (33.01%). Cases had a lower median intake of vitamin C and fewer of them were in the highest quartiles of intake compared with controls (Table 1).

**Table 1:** Characteristics of individuals included in the analysis

Characteristics	Cases, *n* (%) *n* = 5362	Controls, *n* (%) *n* = 11,497	*p*-value^a^
**Study label [ref]**			
Italy 1 [18]	230 (4.29)	547 (4.76)	
Italy 2 [19]	1016 (18.95)	1159 (10.08)	
Greece [22]	110 (2.05)	100 (0.87)	
Russia [23]	450 (8.39)	611 (5.31)	
Iran [27]	286 (5.33)	304 (2.64)	
China [25]	711 (13.26)	711 (6.18)	
Portugal [24]	692 (12.91)	1667 (14.50)	
Spain 1 [20]	441 (8.22)	3440 (29.92)	
Spain 2 [21]	401 (7.48)	455 (3.96)	
Mexico 1 [29]	248 (4.63)	478 (4.16)	
Mexico 2 [30]	220 (4.10)	752 (6.54)	
Mexico 3 [31]	234 (4.36)	468 (4.07)	
Japan [26]	153 (2.85)	303 (2.64)	
USA [28]	170 (3.17)	502 (4.37)	
**Type of study**			
Clinic-based	1578 (29.43)	2484 (21.61)	
Community-based	3784 (70.57)	9013 (78.39)	
**Sex**			< 0.0001
Male	3318 (61.88)	6282 (54.64)	
Female	2044 (38.12)	5215 (45.36)	
**Age, years**			< 0.0001
≤ 40	278 (5.18)	733 (6.38)	
41–50	599 (11.17)	1648 (14.33)	
51–60	1087 (20.27)	2459 (21.39)	
61–70	1822 (33.98)	3685 (32.05)	
> 70	1576 (29.39)	2968 (25.82)	
Missing	0 (0.00)	4 (0.03)	
**Socioeconomic status**			< 0.0001
Low	3072 (57.29)	5249 (45.66)	
Intermediate	1661 (30.98)	3877 (33.72)	
High	541 (10.09)	2229 (19.39)	
Missing	88 (1.64)	142 (1.24)	
**BMI**			< 0.0001
Underweight (< 18.5 kg/m^2^)	140 (2.61)	129 (1.12)	
Normal weight (18.5–24.9 kg/m^2^)	1929 (35.98)	3345 (29.09)	
Overweight (25–29.9 kg/m^2^)	1396 (26.04)	3534 (30.74)	
Obese (≥ 30 kg/m^2^)	1144 (21.34)	2191 (19.06)	
Missing	753 (14.04)	2298 (19.99)	
**Tobacco smoking status**			0.002
Never	2505 (46.72)	5547 (48.25)	
Former	1325 (24.71)	2976 (25.89)	
Current	1425 (26.58)	2775 (24.14)	
Missing	107 (2.00)	199 (1.73)	
**Alcohol drinking status**			0.189
Never	1744 (32.53)	3482 (30.29)	
Ever	3477 (64.85)	7277 (63.29)	
Missing	141 (2.63)	738 (6.42)	
**Subsite**			
Cardia	495 (9.23)		
Noncardia	3058 (57.03)		
Missing	1809 (33.74)		
**Histological type**			
Intestinal	1770 (33.01)		
Diffuse	1159 (21.62)		
Other/mixed/undifferentiated/ unclassified	815 (15.20)		
Missing	1618 (30.18)		
***H. pylori*** **seropositivity**			0.022
Negative	403 (7.52)	975 (8.48)	
Positive	1598 (29.80)	4,498 (39.12)	
Missing	3361 (62.68)	6,024 (52.40)	
**Intake of fruit and vegetables**			< 0.0001
Low	1726 (32.19)	2,991 (26.02)	
Intermediate	1592 (29.69)	3,480 (30.27)	
High	1620 (30.21)	3,807 (33.11)	
Missing	424 (7.91)	1,219 (10.60)	
**Dietary intake of vitamin C (mg/day), median (IQR)**	89.55 (99.52)	121.02 (113.62)	< 0.0001
**Dietary intake of vitamin C (mg/day), quartile**			< 0.0001
Quartile 1	1596 (29.77)	2745 (23.88)	
Quartile 2	1301 (24.26)	2764 (24.04)	
Quartile 3	1152 (21.48)	2768 (24.08)	
Quartile 4	1143 (21.32)	2748 (23.90)	
Missing	170 (3.17)	472 (4.11)	

*BMI* body mass index, *GC* gastric cancer, *IQR* interquartile range

Missing values include individuals from studies with information on the variable not available/not collected

^a^From *χ*^2^ test or Mann–Whitney test, as appropriate; missing values excluded from the computation

Estimates of the association between dietary vitamin C and GC are reported in Table 2. The ORs were 0.78 (95% CI: 0.71, 0.86) in quartile 2, 0.69 (95% CI: 0.62, 0.76) in quartile 3 and 0.64 (95% CI: 0.58, 0.72) in quartile 4 of intake compared with quartile 1. However, when additionally adjusting for BMI and intake of fruit and vegetables, the observed association was attenuated and the OR for the highest versus lowest quartile of dietary vitamin C was 0.85 (95% CI: 0.73, 0.98). The E-value for the OR was 1.64, while it was 1.15 for the upper limit of its 95% CI (Supplementary Fig. 2). Exclusion of individuals in the highest and lowest 1% of intake or modelling total energy intake using fractional polynomials did not substantially modify results (Table 2).

**Table 2:** Pooled adjusted odds ratios (ORs) and corresponding 95% confidence intervals (CIs) for the association between dietary intake of vitamin C and gastric cancer

	Quartile 1 (ref)		Quartile 2			Quartile 3			Quartile 4			
Model	*n* cases	*n* controls	*n* cases	*n* controls	OR (95% CI)	*n* cases	*n* controls	OR (95% CI)	*n* cases	*n* controls	OR (95% CI)	*P* for trend
Model 1	1596	2744	1301	2763	0.78 (0.71, 0.86)	1152	2766	0.69 (0.62, 0.76)	1143	2748	0.64 (0.58, 0.72)	< 0.0001
Model 2	1525	2627	1251	2646	0.89 (0.79, 0.99)	1092	2649	0.83 (0.73, 0.94)	1090	2631	0.85 (0.73, 0.98)	0.019
Model 3	1458	2538	1251	2646	0.87 (0.78, 0.98)	1092	2649	0.81 (0.72, 0.93)	1053	2513	0.82 (0.71, 0.95)	0.006
Model 4	1525	2627	1251	2646	0.88 (0.79, 0.98)	1092	2649	0.82 (0.72, 0.93)	1090	2631	0.84 (0.72, 0.97)	0.012

Model 1: adjusted for sex (male, female), age (≤ 40 years, 41–50 years, 51–60 years, 61–70 years,  > 70 years, missing), socioeconomic status (low, intermediate, high, missing), tobacco smoking status (never, former, current, missing), alcohol drinking status (never, ever, missing), total energy intake (continuous)

Model 2: same as model 1, and additionally adjusted for body mass index (underweight, normal weight, overweight, obese, missing), intake of fruit and vegetables (low, intermediate, high, missing)

Model 3: same as model 2, individuals in the highest and lowest 1% of vitamin C intake excluded

Model 4: same as model 2, total energy intake modelled using a second-order fractional polynomial

A consistent inverse association across all considered quartiles of vitamin C was found for noncardia GC, while results were not significant for cardia GC (Table 3). As for histological types, individuals in quartile 4 of dietary vitamin C had reduced OR of both diffuse and intestinal types of GC compared with participants in quartile 1 (Table 3).

**Table 3:** Pooled adjusted odds ratios (ORs) and corresponding 95% confidence intervals (CIs) for the association between dietary intake of vitamin C and gastric cancer, by subsite and histological type

Stratum	Quartile 1 (ref)		Quartile 2			Quartile 3			Quartile 4		
	*n* cases	*n* controls	*n* cases	*n* controls	OR (95% CI)	*n* cases	*n* controls	OR (95% CI)	*n* cases	*n* controls	OR (95% CI)
**Subsite**											
Cardia	171	2627	103	2646	0.73 (0.55, 0.97)	95	2649	0.77 (0.55, 1.08)	98	2631	0.86 (0.58, 1.28)
Noncardia	959	2627	756	2646	0.81 (0.71, 0.93)	634	2649	0.69 (0.59, 0.81)	586	2631	0.63 (0.52, 0.76)
**Histological type**											
Intestinal	548	2627	442	2646	0.82 (0.70, 0.97)	325	2649	0.62 (0.50, 0.76)	317	2631	0.61 (0.48, 0.78)
Diffuse	290	2627	239	2646	0.78 (0.63, 0.97)	256	2649	0.79 (0.62, 1.01)	210	2631	0.62 (0.46, 0.83)
Other	256	2627	195	2646	0.84 (0.66, 1.06)	169	2649	0.80 (0.61, 1.04)	182	2631	0.90 (0.65, 1.23)

Analyses were adjusted for sex (male, female), age (≤ 40 years, 41–50 years, 51–60 years, 61–70 years,  > 70 years, missing), socioeconomic status (low, intermediate, high, missing), tobacco smoking status (never, former, current, missing), alcohol drinking status (never, ever, missing), body mass index (underweight, normal weight, overweight, obese, missing), intake of fruit and vegetables (low, intermediate, high, missing), total energy intake (continuous)

Findings of the stratified analyses are reported in Table 4. An inverse association between vitamin C and GC was observed for all considered quartiles of intake among clinic-based studies, but not among community-based ones. In addition, estimates showed non-significant associations across strata of both age and sex. Instead, considering the highest versus lowest quartiles of intake, an inverse association between vitamin C and GC, was observed among individuals reporting low (OR: 0.81; 95% CI: 0.66, 0.99) or high socioeconomic status (OR: 0.48; 95% CI: 0.29, 0.77), while a lack of association was observed among those with moderate socioeconomic status. Also, the association was significant among participants with normal weight and among overweight individuals. Quartile 4 of vitamin C intake was inversely associated with GC among never and former smokers and ever drinkers, while no association was found among other strata of tobacco smoking and alcohol drinking (Table 4). Additionally, vitamin C was found to be inversely associated with GC among individuals both with and without *H. pylori* seropositivity, with similar results when comparing all cases to seropositive controls. However, no significant heterogeneity across considered strata was detected.

**Table 4:** Pooled adjusted odds ratios (ORs) and corresponding 95% confidence intervals (CIs) for the association between dietary intake of vitamin C and gastric cancer, stratified by selected participants’ characteristics

	Quartile 1 (ref)		Quartile 2			Quartile 3			Quartile 4			
Stratum	*n* cases	*n* controls	*n* cases	*n* controls	OR (95% CI)	*n* cases	*n* controls	OR (95% CI)	*n* cases	*n* controls	OR (95% CI)	*p*_heterogeneity_
**Type of study**												0.583
Clinic-based	401	491	341	511	0.78 (0.62, 0.97)	280	515	0.64 (0.49, 0.83)	319	499	0.70 (0.53, 0.94)	
Community-based	1124	2136	910	2135	0.92 (0.81, 1.05)	812	2134	0.92 (0.79, 1.06)	771	2132	0.93 (0.78, 1.10)	
**Sex**												0.887
Male	973	1534	800	1511	0.88 (0.77, 1.02)	670	1419	0.83 (0.70, 0.97)	658	1325	0.89 (0.73, 1.07)	
Female	552	1093	451	1135	0.88 (0.74, 1.06)	422	1230	0.84 (0.69, 1.03)	432	1306	0.81 (0.64, 1.03)	
**Age**												0.500
≤ 60 years	524	1032	440	1095	0.83 (0.69, 0.99)	407	1155	0.79 (0.64, 0.97)	441	1175	0.82 (0.64, 1.04)	
> 60 years	1001	1595	811	1551	0.92 (0.81, 1.06)	685	1494	0.87 (0.74, 1.02)	649	1456	0.89 (0.74, 1.08)	
**Socioeconomic status**												0.211
Low	1011	1303	728	1222	0.87 (0.76, 1.01)	593	1185	0.81 (0.68, 0.96)	546	1102	0.81 (0.66, 0.99)	
Intermediate	385	884	392	851	1.08 (0.88, 1.32)	380	917	1.01 (0.81, 1.26)	384	913	1.05 (0.81, 1.36)	
High	112	403	109	513	0.59 (0.40, 0.86)	106	507	0.48 (0.31, 0.74)	137	577	0.48 (0.29, 0.77)	
**BMI**												0.526
Underweight	47	35	35	32	1.01 (0.35, 2.91)	28	27	0.92 (0.26, 3.22)	27	27	1.79 (0.40, 8.10)	
Normal weight	655	776	453	789	0.71 (0.59, 0.87)	402	852	0.57 (0.46, 0.72)	389	811	0.53 (0.41, 0.70)	
Overweight	379	817	364	867	0.89 (0.73, 1.10)	300	833	0.80 (0.62, 1.02)	296	851	0.75 (0.56, 0.996)	
Obese	303	551	269	493	1.14 (0.90, 1.45)	259	510	1.09 (0.84, 1.41)	274	525	1.25 (0.93, 1.69)	
**Tobacco smoking status**												0.110
Never	716	1183	565	1244	0.88 (0.75, 1.03)	527	1312	0.85 (0.71, 1.02)	491	1362	0.80 (0.64, 0.99)	
Former	338	696	321	677	0.89 (0.71, 1.12)	282	703	0.69 (0.53, 0.90)	263	668	0.60 (0.44, 0.82)	
Current	445	710	339	694	0.85 (0.69, 1.05)	276	606	0.90 (0.70, 1.14)	318	573	1.19 (0.91, 1.58)	
**Alcohol drinking status**												0.921
Never	535	836	391	773	1.02 (0.84, 1.24)	345	789	1.03 (0.82, 1.28)	347	851	1.02 (0.79, 1.32)	
Ever	983	1704	857	1801	0.83 (0.72, 0.95)	744	1799	0.74 (0.63, 0.86)	738	1719	0.76 (0.63, 0.91)	
***H. pylori*** **seropositivity**												0.619
Negative	95	190	94	216	0.58 (0.36, 0.91)	84	222	0.58 (0.34, 0.96)	85	218	0.47 (0.26, 0.85)	
Positive	469	933	315	960	0.78 (0.63, 0.96)	293	998	0.76 (0.59, 0.97)	259	1019	0.66 (0.50, 0.89)	
All cases vs. positive controls	695	933	516	960	0.81 (0.68, 0.98)	477	998	0.80 (0.65, 0.99)	419	1019	0.69 (0.54, 0.89)	

Analyses were adjusted for sex (male, female), age (≤ 40 years, 41–50 years, 51–60 years, 61–70 years,  > 70 years, missing), socioeconomic status (low, intermediate, high, missing), tobacco smoking status (never, former, current, missing), alcohol drinking status (never, ever, missing), body mass index (underweight, normal weight, overweight, obese, missing), intake of fruit and vegetables (low, intermediate, high, missing), total energy intake (continuous)

Findings of the interaction analysis are reported in Supplementary Table 3. The only significant results were found for BMI, which showed a positive additive interaction with vitamin C. Indeed, participants with normal weight and with an intake of vitamin C lower than the median had the strongest association with GC among the considered joint strata (OR: 1.80; 95% CI: 1.56, 2.06), with 19% (95% CI: 8%, 31%) of GC cases among these doubly exposed individuals being attributable to interaction.

**Fig. 1:**
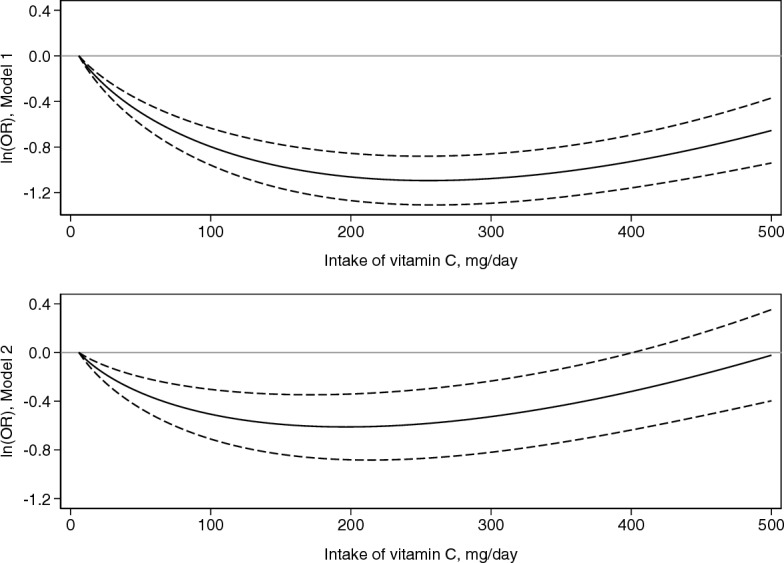
Dose–response relationship between dietary intake of vitamin C and gastric cancer fitted by using one-stage logistic mixed-effects models with second-order fractional polynomial. Solid black line: ln(odds ratio, OR), dashed black line: 95% confidence interval, solid horizontal grey line: OR = 1. Model 1 (*n* = 15,888): adjusted for sex (male, female), age (≤ 40 years, 41–50 years, 51–60 years, 61–70 years,  > 70 years, missing), socioeconomic status (low, intermediate, high, missing), tobacco smoking status (never, former, current, missing), alcohol drinking status (never, ever, missing), total energy intake (continuous). Model 2 (*n* = 15,200): adjusted for sex (male, female), age (≤ 40 years, 41–50 years, 51–60 years, 61–70 years,  > 70 years, missing), socioeconomic status (low, intermediate, high, missing), tobacco smoking status (never, former, current, missing), alcohol drinking status (never, ever, missing), body mass index (underweight, normal weight, overweight, obese, missing), intake of fruit and vegetables (low, intermediate, high, missing), total energy intake (continuous). Reference value of dietary vitamin C: 6 mg/day

In line with the results of the overall analysis, the dose–response models showed attenuated estimates of ORs when adjusting for BMI and intake of fruit and vegetables (Fig. 1, Table 5), with ORs decreasing up to 150–200 mg/day of vitamin C (OR: 0.54; 95% CI: 0.41, 0.71). For higher intakes, instead, ORs tended to increase towards unity, which was included in the 95% CI for values of dietary vitamin C of 400 mg/day or higher.

**Table 5:** Results of the dose–response analysis on dietary intake of vitamin C and gastric cancer fitted by using one-stage logistic mixed-effects models with second-order fractional polynomial

	Model 1 (*n* = 15,888)	Model 2 (*n* = 15,200)
	ln(OR) = − 0.93 intake^1^ + 0.48 intake^1^	ln(OR) = − 0.60 intake^1^ + 0.36 intake^1^
Vitamin C, mg/day	OR^a^ (95% CI)	OR^a^ (95% CI)
50	0.61 (0.55, 0.68)	0.72 (0.63, 0.82)
100	0.45 (0.38, 0.53)	0.60 (0.49, 0.74)
150	0.38 (0.31, 0.46)	0.55 (0.43, 0.71)
200	0.35 (0.28, 0.43)	0.54 (0.41, 0.71)
250	0.34 (0.27, 0.41)	0.56 (0.42, 0.74)
300	0.34 (0.27, 0.42)	0.59 (0.44, 0.79)
350	0.36 (0.29, 0.45)	0.65 (0.48, 0.87)
400	0.40 (0.31, 0.50)	0.73 (0.53, 1.00)
450	0.45 (0.35, 0.58)	0.83 (0.59, 1.17)
500	0.52 (0.39, 0.69)	0.98 (0.67, 1.42)

*OR* odds ratio, *CI* confidence interval

Model 1: adjusted for sex (male, female), age (≤ 40 years, 41–50 years, 51–60 years, 61–70 years,  > 70 years, missing), socioeconomic status (low, intermediate, high, missing), tobacco smoking status (never, former, current, missing), alcohol drinking status (never, ever, missing), total energy intake (continuous)

Model 2: adjusted for sex (male, female), age (≤ 40 years, 41–50 years, 51–60 years, 61–70 years,  > 70 years, missing), socioeconomic status (low, intermediate, high, missing), tobacco smoking status (never, former, current, missing), alcohol drinking status (never, ever, missing), body mass index (underweight, normal weight, overweight, obese, missing), intake of fruit and vegetables (low, intermediate, high, missing), total energy intake (continuous)

^a^Reference value of dietary vitamin C: 6 mg/day

## Discussion

Our pooled analysis of individual-level data suggests that dietary vitamin C is inversely associated with GC and, even though its strength was attenuated, this association persisted after adjustment for intake of fruit and vegetables. This relationship was limited to noncardia GC, with no significant association for cardia GC, while the results were similar for intestinal and diffuse types of GC. Although estimates showed occasional slight differences across strata of patients’ characteristics, no significant heterogeneity was found, suggesting that the association between vitamin C and GC does not substantially vary according to considered strata. However, the findings of the interaction analysis for BMI also suggest that individuals who would benefit the most from an increased intake of vitamin C are those with normal weight. This result could be either due to both a healthier diet among individuals who are neither overweight nor obese or to reverse causality, since GC might lead to weight loss itself [38, 39]. Lastly, the dose–response analysis indicates that dietary levels of vitamin C higher than the recommended intake, which is equal to 90 mg/day for men and 75 mg/day for women [40], and up to 150/200 mg/day could be beneficial for the reduction of GC risk.

Three recent meta-analyses reported an inverse association between dietary intake of vitamin C and GC, in line with our results [13–15]. In contrast with our findings, however, Li et al. found that this was consistent among both clinic- and community-based studies, while a non-significant result was observed for diffuse type GC [15]. In addition, some differences in their results compared with our findings might also be explained by differences in levels of intake of vitamin C between the study populations. A few additional studies have been published after those meta-analyses [41, 42], further confirming the inverse relationship between dietary vitamin C and GC. Moreover, Kim et al. reported that vitamin C was inversely associated with GC only among nonsmokers [41], in line with our results suggesting an association only among never and former smokers.

Our study was conducted using data from several case–control studies carried out in different geographic regions and which were harmonized centrally at the StoP coordinating center using a common methodology. In addition, we were able to adjust the analyses for a number of relevant covariates, including intake of fruit and vegetables that has been overlooked by most of previous epidemiological studies [13–15]. Also, the large sample size allowed us to investigate whether the relationship between dietary vitamin C and GC was consistent across strata of participants’ characteristics, which could help to identify individuals who would benefit the most from related nutritional interventions. With the same aim, we assessed the occurrence of additive interactions, which are commonly deemed more relevant at informing public health interventions compared with multiplicative interactions [35], between vitamin C and other selected risk or protective factors.

Among limitations, our analysis relied on previously conducted studies which were based on different methodologies, including the adoption of different FFQs used for exposure assessment together with country-specific databases, possibly contributing to heterogeneity between included studies and to some discrepancies with previous meta-analyses, as described above. In addition, studies contributing data to our analyses had a case–control design, which may suffer from selection bias, in particular among clinic-based studies [43, 44]. Furthermore, since dietary information was self-reported through questionnaires, exposure could be affected by recall bias, although it has been reported to be limited in case–control studies investigating micronutrients [45]. Similarly, information on relevant covariates, such as tobacco smoking, alcohol drinking, BMI, and others, might be affected by recall bias too. As for BMI, in particular, results of the interaction analysis might be biased also because individuals with GC might have undergone weight loss due to the disease itself before the actual diagnosis caused by tumor-related obstruction of the upper digestive tract, malabsorption, anorexia, and hypermetabolism [38, 39]. Thus, leading to apparent lower OR of GC among overweight or obese individuals. Moreover, evaluation of *H. pylori* serostatus has been reported to lead to misclassification of previous infection, differentially among cases and controls [46, 47], although this issue was partially addressed by the sensitivity analysis we carried out where we considered all cases as seropositive. Additionally, although the association between vitamin C and GC was still significant after adjustment for intake of fruit and vegetables, this finding warrants further investigation to rule out whether the observed relationship is due to a real effect of vitamin C or to other factors including nutrients that can be found in fruit and vegetables, and thus to various sources of bias, such as residual and unmeasured confounding. Indeed, since dietary vitamin C mostly derives from intake of fruit and vegetables, their intakes are typically related, hence making it difficult to separate the effect of vitamin C from that of other components present in fruit and vegetables, such as other antioxidants.

In conclusion, the results of the present analysis carried out by pooling data from fourteen epidemiological studies suggest that vitamin C is inversely associated with GC. However, further well-designed prospective studies, aimed at disentangling the complex relationships between intake of fruit and vegetables, vitamins and other antioxidants, and GC, are warranted to prove causality of the observed relationship between vitamin C and GC.

### Supplementary Information

Below is the link to the electronic supplementary material.

Supplementary file1 (PDF 567 KB)

## Data Availability

Data described in the manuscript, code book, and analytic code will be made available upon request from the corresponding author pending approval of the participating study centers of the StoP Project.

## Abbreviations

AP: Attributable proportion
BMI: Body mass index
CI: Confidence interval
FFQ: Food-frequency questionnaire
GC: Gastric cancer
*H. pylori*: *Helicobacter pylori*
OR: Odds ratio
RERI: Relative excess risk due to interaction
StoP Project: Stomach cancer Pooling Project
