# Supplementary material for: Dietary intake of vitamin C and gastric cancer: a pooled analysis within the Stomach cancer Pooling (StoP) Project
Source: Gastric Cancer. 2024 Mar 4;27(3):461–72. doi: 10.1007/s10120-024-01476-8 (PMC11016516; doi:10.1007/s10120-024-01476-8)
Supplement: Supplementary file 1 — Supplementary file1 (PDF 567 KB) [file 10120_2024_1476_MOESM1_ESM.pdf]

Dietary intake of vitamin C and gastric cancer: a pooled analysis within the Stomach cancer Pooling (StoP) Project

Michele Sassano[1], Monireh Sadat Seyyedsalehi[1], Giulia Collatuzzo[1], Claudio Pelucchi[2], Rossella Bonzi[2], Monica Ferraroni[2], Domenico Palli[3], Guo-Pei Yu[4], Zuo-Feng Zhang[5], Lizbeth López-Carrillo[6], Nuno Lunet[7,8,9], Samantha Morais[7,8,9], David Zaridze[10], Dmitry Maximovich[10], Vicente Martín[11,12], Gemma Castano-Vinyals[11,13,14,15], Jesús Vioque[11,16], Sandra González-Palacios[11,16], Mary H. Ward[17], Reza Malekzadeh[18], Mohammadreza Pakseresht[18,19,20], Raul Ulises Hernández-Ramirez[21], Malaquias López-Cervantes[22], Eva Negri[1,2], Federica Turati[2], Charles S. Rabkin[17], Shoichiro Tsugane[23,24], Akihisa Hidaka[25,26], Areti Lagiou[27], Pagona Lagiou[28,29], M. Constanza Camargo[17], Maria Paula Curado[30], Carlo La Vecchia[2], Paolo Boffetta[1,31,32]*

1. Department of Medical and Surgical Sciences, University of Bologna, Bologna, Italy.
2. Department of Clinical Sciences and Community Health, University of Milan, Milan, Italy.
3. Cancer Risk Factors and Life-Style Epidemiology Unit, Institute for Cancer Research, Prevention and Clinical Network, ISPRO, Florence, Italy.
4. Medical Informatics Center, Peking University, Beijing, China.
5. Department of Epidemiology, UCLA Fielding School of Public Health and Jonsson Comprehensive Cancer Center, Los Angeles, CA, USA.
6. Mexico National Institute of Public Health, Cuernavaca, Mexico.
7. EPIUnit, Instituto de Saúde Pública da Universidade do Porto, Porto, Portugal.
8. Laboratório para a Investigação Integrativa e Translacional em Saúde Populacional (ITR), Porto, Portugal.
9. Departamento de Ciências da Saúde Pública e Forenses e Educação Médica, Faculdade de Medicina, Universidade do Porto, Porto, Portugal.
10. Department of Clinical Epidemiology, N.N. Blokhin National Medical Research Center for Oncology, Moscow, Russia.
11. Consortium for Biomedical Research in Epidemiology and Public Health (CIBERESP), Madrid, Spain.
12. Instituto de Biomedicina, Universidad de León, León, Spain.
13. Barcelona Institute for Global Health-ISGlobal, Barcelona, Spain.
14. IMIM (Hospital del Mar Medical Research Institute), Barcelona, Spain.
15. Universitat Pompeu Fabra (UPF), Barcelona, Spain.
16. Instituto de Investigación Sanitaria y Biomédica de Alicante, Universidad Miguel Hernandez (ISABIAL-UMH), Alicante, Spain.

17. Division of Cancer Epidemiology and Genetics, National Cancer Institute, National Institutes of Health, Rockville, MD, USA.
18. Digestive Oncology Research Center, Digestive Disease Research Institute, Tehran University of Medical Sciences, Tehran, Iran.
19. Department of Agricultural, Food and Nutritional Sciences, University of Alberta, Edmonton, Canada.
20. Nutritional Epidemiology Group, Centre for Epidemiology and Biostatistics, University of Leeds, Leeds, UK.
21. Department of Biostatistics, Yale School of Public Health, New Haven, CT, USA.
22. Facultad de Medicina, National Autonomous University of Mexico (UNAM), Coyoacán, Mexico.
23. Division of Cohort Research, National Cancer Center Institute for Cancer Control, Tokyo, Japan.
24. International University of Health and Welfare Graduate School of Public Health, Tokyo, Japan.
25. Division of Epidemiology, National Cancer Center Institute for Cancer Control, Tokyo, Japan.
26. Department of Diabetes and Endocrinology, JCHO Tokyo Yamate Medical Centre, Tokyo, Japan.
27. Department of Public and Community Health, School of Public Health, University of West Attica, Athens, Greece.
28. Department of Hygiene, Epidemiology and Medical Statistics, School of Medicine, National and Kapodistrian University of Athens, Athens, Greece.
29. Department of Epidemiology, Harvard T.H. Chan School of Public Health, Boston, MA, USA.
30. Centro Internacional de Pesquisa, A. C. Camargo Cancer Center, São Paulo, Brasil.
31. Stony Brook Cancer Center, Stony Brooke University, Stony Brook, NY, USA.
32. Department of Family, Population and Preventive Medicine, Renaissance School of Medicine, Stony Brook University, Stony Brook, NY, USA.

*Correspondence:
Paolo Boffetta
Department of Medical and Surgical Sciences
University of Bologna
Via Massarenti 9 - Bologna 40138, Italy
paolo.boffetta@unibo.it

Supplementary Table 1. Main characteristics of the studies included in the analysis.

Supplementary Table 2. Study participants' characteristics, by study included in the analysis.

Supplementary Table 3. Results of the interaction analysis between dietary intake of vitamin C and other selected factors.

Supplementary Figure 1. Flowchart describing inclusion of studies and participants.

Supplementary Figure 2. E-value for the point estimate and the upper limit of the 95% confidence interval for the observed association between dietary intake of vitamin C and gastric cancer.

Supplementary Table 1. Main characteristics of the studies included in the analysis.

Study label [ref]	Type of study	Study period	n cases, by quartile (Q) of intake of vitamin C	n controls, by quartile (Q) of intake of vitamin C
Italy 1 [18]	Clinic-based	1997-2007	Q1: 57<br>Q2: 63<br>Q3: 63<br>Q4: 47	Q1: 137<br>Q2: 137<br>Q3: 137<br>Q4: 136
Italy 2 [19]	Community-based	1985-1987	Q1: 335<br>Q2: 272<br>Q3: 208<br>Q4: 201	Q1: 290<br>Q2: 292<br>Q3: 289<br>Q4: 288
Greece [22]	Clinic-based	1981-1984	Q1: 47<br>Q2: 14<br>Q3: 16<br>Q4: 33	Q1: 25<br>Q2: 25<br>Q3: 25<br>Q4: 25
Russia [23]	Clinic-based	1996-1997	Q1: 109<br>Q2: 118<br>Q3: 111<br>Q4: 112	Q1: 153<br>Q2: 153<br>Q3: 153<br>Q4: 152
Iran [27]	Community-based	2005-2007	Q1: 153<br>Q2: 75<br>Q3: 32<br>Q4: 24	Q1: 76<br>Q2: 76<br>Q3: 76<br>Q4: 76
China [25]	Community-based	1991-1993	Q1: 171<br>Q2: 168<br>Q3: 173<br>Q4: 195	Q1: 177<br>Q2: 176<br>Q3: 177<br>Q4: 176
Portugal [24]	Community-based	1999-2006	Q1: 259<br>Q2: 155<br>Q3: 144<br>Q4: 75	Q1: 400<br>Q2: 400<br>Q3: 400<br>Q4: 400
Spain 1 [20]	Community-based	2008-2012	Q1: 94<br>Q2: 81<br>Q3: 85<br>Q4: 79	Q1: 760<br>Q2: 760<br>Q3: 760<br>Q4: 760
Spain 2 [21]	Clinic-based	1995-1999	Q1: 136<br>Q2: 114<br>Q3: 62<br>Q4: 86	Q1: 114<br>Q2: 114<br>Q3: 114<br>Q4: 113
Mexico 1 [29]	Community-based	2004-2005	Q1: 28<br>Q2: 55<br>Q3: 77<br>Q4: 88	Q1: 120<br>Q2: 119<br>Q3: 120<br>Q4: 119
Mexico 2 [30]	Community-based	1989-1990	Q1: 39<br>Q2: 65<br>Q3: 49<br>Q4: 67	Q1: 188<br>Q2: 188<br>Q3: 188<br>Q4: 188
Mexico 3 [31]	Clinic-based	1994-1996	Q1: 71<br>Q2: 50<br>Q3: 60<br>Q4: 53	Q1: 117<br>Q2: 117<br>Q3: 117<br>Q4: 117
Japan 3 [26]	Clinic-based	1998-2002	Q1: 52<br>Q2: 32<br>Q3: 28<br>Q4: 41	Q1: 62<br>Q2: 82<br>Q3: 86<br>Q4: 73
USA [28]	Community-based	1988-1993	Q1: 45<br>Q2: 39<br>Q3: 44<br>Q4: 42	Q1: 126<br>Q2: 125<br>Q3: 126<br>Q4: 125

Supplementary Table 2. Study participants' characteristics, by study included in the analysis.

	Study																											
	Italy 1		Italy 2		Greece		Russia		Iran		China		Portugal		Spain 1		Spain 2		Mexico 1		Mexico 2		Mexico 3		Japan		USA	
Characteristics	Cases, n (%) n=230	Controls, n (%) n=547	Cases, n (%) n=1,016	Controls, n (%) n=1,159	Cases, n (%) n=110	Controls, n (%) n=100	Cases, n (%) n=450	Controls, n (%) n=611	Cases, n (%) n=286	Controls, n (%) n=304	Cases, n (%) n=711	Controls, n (%) n=711	Cases, n (%) n=692	Controls, n (%) n=1,667	Cases, n (%) n=441	Controls, n (%) n=3,440	Cases, n (%) n=401	Controls, n (%) n=455	Cases, n (%) n=248	Controls, n (%) n=478	Cases, n (%) n=220	Controls, n (%) n=752	Cases, n (%) n=234	Controls, n (%) n=468	Cases, n (%) n=153	Controls, n (%) n=303	Cases, n (%) n=170	Controls, n (%) n=502
**Sex**																												
Male	143 (62.2)	286 (52.3)	640 (63.0)	705 (60.8)	57 (51.8)	49 (49.0)	248 (55.1)	293 (48.0)	210 (73.4)	217 (71.4)	453 (63.7)	453 (63.7)	417 (60.3)	783 (47.0)	290 (65.8)	1,892 (55.0)	266 (66.3)	285 (62.6)	134 (54.0)	258 (54.0)	122 (55.5)	296 (39.4)	133 (56.8)	266 (56.8)	108 (70.6)	215 (71.0)	97 (57.1)	284 (56.6)
Female	87 (37.8)	261 (47.7)	376 (37.0)	454 (39.2)	53 (48.2)	51 (51.0)	202 (44.9)	318 (52.1)	76 (26.6)	87 (28.6)	258 (36.3)	258 (36.3)	275 (39.7)	884 (53.0)	151 (34.2)	1,548 (45.0)	135 (33.7)	170 (37.4)	114 (46.0)	220 (46.0)	98 (44.5)	456 (60.6)	101 (43.2)	202 (43.2)	45 (29.4)	88 (29.0)	73 (42.9)	218 (43.4)
**Age, years**																												
≤40	11 (4.8)	28 (5.1)	25 (2.5)	39 (3.4)	7 (6.4)	6 (6.0)	12 (2.7)	61 (10.0)	8 (2.8)	4 (1.3)	49 (6.9)	50 (7.0)	36 (5.2)	154 (9.2)	14 (3.2)	100 (2.9)	10 (2.5)	13 (2.9)	35 (14.1)	64 (13.4)	32 (14.6)	86 (11.4)	30 (12.8)	63 (13.5)	7 (4.6)	18 (5.9)	2 (1.2)	47 (9.4)
41-50	33 (14.4)	83 (15.2)	90 (8.9)	102 (8.8)	9 (8.2)	19 (19.0)	56 (12.4)	160 (26.2)	16 (5.6)	43 (14.1)	59 (8.3)	62 (8.7)	105 (15.2)	372 (22.3)	34 (7.7)	405 (11.8)	44 (11.0)	53 (11.7)	39 (15.7)	67 (14.0)	40 (18.2)	113 (15.0)	37 (15.8)	73 (15.6)	30 (19.6)	49 (16.2)	7 (4.1)	47 (9.4)
51-60	58 (25.2)	134 (24.5)	213 (21.0)	240 (20.7)	18 (16.4)	25 (25.0)	109 (24.2)	175 (28.6)	57 (19.9)	77 (25.3)	141 (19.8)	130 (18.3)	130 (18.8)	363 (21.8)	90 (20.4)	652 (19.0)	70 (17.5)	97 (21.3)	55 (22.2)	114 (23.9)	38 (17.3)	182 (24.2)	53 (22.7)	114 (24.4)	44 (28.8)	103 (34.0)	11 (6.5)	53 (10.6)
61-70	89 (38.7)	210 (38.4)	397 (39.1)	432 (37.3)	41 (37.3)	30 (30.0)	188 (41.8)	158 (25.9)	88 (30.8)	92 (30.3)	271 (38.1)	293 (41.2)	182 (26.3)	437 (26.2)	106 (24.0)	1,174 (34.1)	133 (33.2)	169 (37.1)	75 (30.2)	126 (26.4)	80 (36.4)	214 (28.5)	70 (29.9)	138 (29.5)	70 (45.8)	122 (40.3)	32 (18.8)	90 (17.9)
>70	39 (17.0)	92 (16.8)	291 (28.6)	346 (29.9)	35 (31.8)	20 (20.0)	85 (18.9)	57 (9.3)	117 (40.9)	88 (29.0)	191 (26.9)	176 (24.8)	239 (34.5)	341 (20.5)	197 (44.7)	1109 (32.2)	144 (35.9)	123 (27.0)	44 (17.7)	107 (22.4)	30 (13.6)	157 (20.9)	44 (18.8)	80 (17.1)	2 (1.3)	11 (3.6)	118 (69.4)	261 (52.0)
Missing	0 (0.0)	0 (0.0)	0 (0.0)	0 (0.0)	0 (0.0)	0 (0.0)	0 (0.0)	0 (0.0)	0 (0.0)	0 (0.0)	0 (0.0)	0 (0.0)	0 (0.0)	0 (0.0)	0 (0.0)	0 (0.0)	0 (0.0)	0 (0.0)	0 (0.0)	0 (0.0)	0 (0.0)	0 (0.0)	0 (0.0)	0 (0.0)	0 (0.0)	0 (0.0)	0 (0.0)	4 (0.8)
**Socioeconomic status**																												
Low	95 (41.3)	236 (43.1)	817 (80.4)	797 (68.8)	91 (82.7)	67 (67.0)	27 (6.0)	25 (4.1)	93 (32.5)	69 (22.7)	478 (67.2)	447 (62.9)	552 (79.8)	752 (45.1)	303 (68.7)	1,802 (52.4)	367 (91.5)	418 (91.9)	61 (24.6)	159 (33.3)	48 (21.8)	200 (26.6)	65 (27.8)	149 (31.8)	58 (37.9)	105 (34.7)	17 (10.0)	23 (4.6)
Intermediate	86 (37.4)	174 (31.8)	167 (16.4)	287 (24.8)	10 (9.1)	6 (6.0)	382 (84.9)	537 (87.9)	159 (55.6)	172 (56.6)	196 (27.6)	228 (32.1)	94 (13.6)	362 (21.7)	91 (20.6)	951 (27.7)	27 (6.7)	30 (6.6)	98 (39.5)	180 (37.7)	77 (35.0)	337 (44.8)	99 (42.3)	205 (43.8)	63 (41.2)	134 (44.2)	112 (65.9)	274 (54.6)
High	46 (20.0)	133 (24.3)	17 (1.7)	61 (5.3)	9 (8.2)	26 (26.0)	36 (8.0)	42 (6.9)	34 (11.9)	63 (20.7)	33 (4.6)	30 (4.2)	37 (5.4)	550 (33.0)	47 (10.7)	683 (19.9)	6 (1.5)	7 (1.5)	89 (35.9)	139 (29.1)	51 (23.2)	149 (19.8)	65 (27.8)	110 (23.5)	31 (20.3)	62 (20.5)	40 (23.5)	174 (34.7)
Missing	3 (1.3)	4 (0.7)	15 (1.5)	14 (1.2)	0 (0.0)	1 (1.0)	5 (1.1)	7 (1.2)	0 (0.0)	0 (0.0)	4 (0.6)	6 (0.8)	9 (1.3)	3 (0.2)	0 (0.0)	4 (0.1)	1 (0.3)	0 (0.0)	0 (0.0)	0 (0.0)	44 (20.0)	66 (8.8)	5 (2.1)	4 (0.9)	1 (0.7)	2 (0.7)	1 (0.6)	31 (6.2)
**Body mass index**																												
Underweight (<18.5 kg/m2)	2 (0.9)	13 (2.4)	28 (2.8)	25 (2.2)	2 (1.8)	1 (1.0)	26 (5.8)	10 (1.6)	9 (3.2)	4 (1.3)	0 (0.0)	0 (0.0)	3 (0.4)	16 (1.0)	3 (0.7)	36 (1.1)	29 (7.2)	8 (1.8)	23 (9.3)	0 (0.0)	0 (0.0)	0 (0.0)	0 (0.0)	0 (0.0)	12 (7.8)	2 (0.7)	3 (1.8)	14 (2.8)
Normal weight (18.5 – 24.9 kg/m2)	116 (50.4)	236 (43.1)	553 (54.4)	580 (50.0)	50 (45.5)	39 (39.0)	210 (46.7)	189 (30.9)	190 (66.4)	106 (34.9)	3 (0.4)	2 (0.3)	211 (30.5)	515 (30.9)	121 (27.4)	1,105 (32.1)	175 (43.6)	126 (27.7)	131 (52.8)	0 (0.0)	0 (0.0)	0 (0.0)	0 (0.0)	0 (0.0)	100 (65.4)	202 (66.7)	69 (40.6)	245 (48.8)
Overweight (25 – 29.9 kg/m2)	82 (35.7)	223 (40.8)	349 (34.4)	452 (39.0)	38 (34.6)	45 (45.0)	101 (22.4)	194 (31.8)	57 (19.9)	125 (41.1)	44 (6.2)	37 (5.2)	255 (36.9)	726 (43.6)	192 (43.5)	1,279 (37.2)	95 (23.7)	181 (39.8)	73 (29.4)	0 (0.0)	0 (0.0)	0 (0.0)	0 (0.0)	0 (0.0)	39 (25.5)	97 (32.0)	71 (41.8)	175 (34.9)
Obese (≥30 kg/m2)	26 (11.3)	73 (13.4)	86 (8.5)	102 (8.8)	15 (13.6)	14 (14.0)	40 (8.9)	106 (17.4)	18 (6.3)	68 (22.4)	659 (92.7)	669 (94.1)	138 (19.9)	383 (23.0)	98 (22.2)	638 (18.6)	19 (4.7)	81 (17.8)	21 (8.5)	0 (0.0)	0 (0.0)	0 (0.0)	0 (0.0)	0 (0.0)	2 (1.3)	2 (0.7)	22 (12.9)	55 (11.0)
Missing	4 (1.7)	2 (0.4)	0 (0.0)	0 (0.0)	5 (4.6)	1 (1.0)	73 (16.2)	112 (18.3)	12 (4.2)	1 (0.3)	5 (0.7)	3 (0.4)	85 (12.3)	27 (1.6)	27 (6.1)	382 (11.1)	83 (20.7)	59 (13.0)	0 (0.0)	478 (100.0)	220 (100.0)	752 (100.0)	234 (100.0)	468 (100.0)	0 (0.0)	0 (0.0)	5 (2.9)	13 (2.6)
**Tobacco smoking status**																												
Never	96 (41.7)	261 (47.7)	359 (35.3)	413 (35.6)	55 (50.0)	49 (49.0)	241 (53.6)	345 (56.5)	172 (60.1)	196 (64.5)	373 (52.5)	415 (58.4)	386 (55.8)	873 (52.4)	184 (41.7)	1,511 (43.9)	165 (41.2)	218 (47.9)	118 (47.6)	232 (48.5)	84 (38.2)	398 (52.9)	133 (56.8)	258 (55.1)	52 (34.0)	148 (48.8)	87 (51.2)	230 (45.8)
Former	71 (30.9)	163 (29.8)	285 (28.1)	381 (32.9)	14 (12.7)	6 (6.0)	73 (16.2)	87 (14.2)	43 (15.0)	45 (14.8)	135 (19.0)	83 (11.7)	162 (23.4)	376 (22.6)	155 (35.2)	1,216 (35.4)	87 (21.7)	114 (25.1)	110 (44.4)	128 (26.8)	0 (0.0)	0 (0.0)	74 (31.6)	115 (24.6)	46 (30.1)	73 (24.1)	70 (41.2)	189 (37.7)
Current	60 (26.1)	122 (22.3)	329 (32.4)	335 (28.9)	41 (37.3)	45 (45.0)	131 (29.1)	177 (29.0)	71 (24.8)	63 (20.7)	202 (28.4)	212 (29.8)	121 (17.5)	361 (21.7)	98 (22.2)	664 (19.3)	149 (37.2)	123 (27.0)	17 (6.9)	104 (21.8)	125 (56.8)	340 (45.2)	21 (9.0)	88 (18.8)	52 (34.0)	78 (25.7)	8 (4.7)	63 (12.6)
Missing	3 (1.3)	1 (0.2)	43 (4.2)	30 (2.6)	0 (0.0)	0 (0.0)	5 (1.1)	2 (0.3)	0 (0.0)	0 (0.0)	1 (0.1)	1 (0.1)	23 (3.3)	57 (3.4)	4 (0.9)	49 (1.4)	0 (0.0)	0 (0.0)	3 (1.2)	14 (2.9)	11 (5.0)	14 (1.9)	6 (2.6)	7 (1.5)	3 (2.0)	4 (1.3)	5 (2.9)	20 (4.0)
**Alcohol drinking status**																												
Never	38 (16.5)	125 (22.9)	132 (13.0)	161 (13.9)	27 (24.6)	45 (45.0)	101 (22.4)	166 (27.2)	278 (97.2)	297 (97.7)	438 (61.6)	452 (63.6)	83 (12.0)	266 (16.0)	58 (13.2)	514 (14.9)	135 (33.7)	178 (39.1)	126 (50.8)	302 (63.2)	91 (41.4)	467 (62.1)	112 (47.9)	225 (48.1)	52 (34.0)	89 (29.4)	73 (42.9)	195 (38.8)
Ever	192 (83.5)	422 (77.2)	884 (87.0)	998 (86.1)	83 (75.5)	55 (55.0)	344 (76.4)	440 (72.0)	8 (2.8)	7 (2.3)	273 (38.4)	255 (35.9)	586 (84.7)	1,344 (80.6)	271 (61.5)	2,262 (65.8)	266 (66.3)	277 (60.9)	122 (49.2)	176 (36.8)	129 (58.6)	285 (37.9)	122 (52.1)	243 (51.9)	101 (66.0)	214 (70.6)	96 (56.5)	299 (59.6)
Missing	0 (0.0)	0 (0.0)	0 (0.0)	0 (0.0)	0 (0.0)	0 (0.0)	5 (1.1)	5 (0.8)	0 (0.0)	0 (0.0)	0 (0.0)	4 (0.6)	23 (3.3)	57 (3.4)	112 (25.4)	664 (19.3)	0 (0.0)	0 (0.0)	0 (0.0)	0 (0.0)	0 (0.0)	0 (0.0)	0 (0.0)	0 (0.0)	0 (0.0)	0 (0.0)	1 (0.6)	8 (1.6)
**Subsite**																												
Cardia	10 (4.4)		69 (6.8)		0 (0.0)		92 (20.4)		65 (22.7)		0 (0.0)		78 (11.3)		95 (21.5)		31 (7.7)		135 (54.4)		0 (0.0)		0 (0.0)		21 (13.7)		34 (20.0)	
Noncardia	92 (40.0)		947 (93.2)		90 (81.8)		201 (44.7)		221 (77.3)		0 (0.0)		546 (78.9)		332 (75.3)		261 (65.1)		111 (44.8)		0 (0.0)		0 (0.0)		126 (82.4)		107 (62.9)	
Missing	128 (55.7)		0 (0.0)		20 (18.2)		157 (34.9)		0 (0.0)		711 (100.0)		68 (9.8)		14 (3.2)		109 (27.2)		2 (0.8)		220 (100.0)		234 (100.0)		6 (3.9)		29 (17.1)	
**Histological type**																												
Intestinal	33 (14.4)		510 (50.2)		0 (0.0)		121 (26.9)		148 (51.8)		0 (0.0)		286 (41.3)		168 (38.1)		243 (60.6)		92 (37.1)		0 (0.0)		80 (34.2)		0 (0.0)		89 (52.4)	
Diffuse	57 (24.8)		216 (21.3)		0 (0.0)		119 (26.4)		68 (23.8)		0 (0.0)		146 (21.1)		103 (23.4)		109 (27.2)		155 (62.5)		0 (0.0)		130 (55.6)		0 (0.0)		56 (32.9)	
Other/mixed/ undifferentiated/ unclassified	64 (27.8)		290 (28.5)		0 (0.0)		210 (46.7)		70 (24.5)		0 (0.0)		94 (13.6)		20 (4.5)		49 (12.2)		1 (0.4)		0 (0.0)		0 (0.0)		0 (0.0)		17 (10.0)	
Missing	76 (33.0)		0 (0.0)		110 (100.0)		0 (0.0)		0 (0.0)		711 (100.0)		166 (24.0)		150 (34.0)		0 (0.0)		0 (0.0)		220 (100.0)		24 (10.3)		153 (100.0)		8 (4.7)	
***H. pylori* seropositivity**																												
Negative	0 (0.0)	0 (0.0)	0 (0.0)	0 (0.0)	0 (0.0)	0 (0.0)	184 (40.9)	226 (37.0)	3 (1.1)	9 (3.0)	0 (0.0)	0 (0.0)	61 (8.8)	148 (8.9)	21 (4.8)	260 (7.6)	0 (0.0)	0 (0.0)	67 (27.0)	112 (23.4)	0 (0.0)	0 (0.0)	40 (17.1)	85 (18.2)	27 (17.6)	135 (44.6)	0 (0.0)	0 (0.0)
Positive	0 (0.0)	0 (0.0)	0 (0.0)	0 (0.0)	0 (0.0)	0 (0.0)	177 (39.3)	215 (35.2)	247 (86.4)	289 (95.1)	0 (0.0)	0 (0.0)	459 (66.3)	1201 (72.1)	252 (57.1)	1,916 (55.7)	0 (0.0)	0 (0.0)	171 (69.0)	355 (74.3)	0 (0.0)	0 (0.0)	166 (70.9)	357 (76.3)	126 (82.4)	165 (54.5)	0 (0.0)	0 (0.0)
Missing	230 (100.0)	547 (100.0)	1,016 (100.0)	1,159 (100.0)	110 (100.0)	100 (100.0)	89 (19.8)	170 (27.8)	36 (12.6)	6 (2.0)	711 (100.0)	711 (100.0)	172 (24.9)	318 (19.1)	168 (38.1)	1,264 (36.7)	401 (100.0)	455 (100.0)	10 (4.0)	11 (2.3)	220 (100.0)	752 (100.0)	28 (12.0)	26 (5.6)	0 (0.0)	3 (1.0)	170 (100.0)	502 (100.0)
**Intake of fruit and vegetables**																												
Low	81 (35.22)	178 (32.54)	388 (38.19)	382 (32.96)	45 (40.91)	28 (28.00)	33 (7.33)	53 (8.67)	146 (51.05)	49 (16.12)	250 (35.16)	212 (29.82)	293 (42.34)	451 (27.05)	108 (24.49)	917 (26.66)	152 (37.91)	133 (29.23)	67 (27.02)	172 (35.98)	96 (43.64)	224 (29.79)	0 (0.0)	0 (0.0)	18 (11.76)	20 (6.60)	49 (28.82)	172 (34.26)
Intermediate	79 (34.35)	181 (33.09)	314 (30.91)	382 (32.96)	31 (28.18)	37 (37.00)	148 (32.89)	184 (30.11)	86 (30.07)	107 (35.20)	227 (31.93)	234 (32.91)	196 (28.32)	549 (32.93)	113 (25.62)	942 (27.38)	137 (34.16)	147 (32.31)	91 (36.69)	156 (32.64)	69 (31.36)	262 (34.84)	0 (0.0)	0 (0.0)	47 (30.72)	124 (40.92)	54 (31.76)	175 (34.86)
High	70 (30.43)	188 (34.37)	314 (30.91)	395 (34.08)	34 (30.91)	35 (35.00)	264 (58.67)	369 (60.39)	52 (18.18)	148 (48.68)	225 (31.65)	250 (35.16)	144 (20.81)	600 (35.99)	108 (24.49)	917 (26.66)	109 (27.18)	175 (38.46)	90 (36.29)	150 (31.38)	55 (25.00)	266 (35.37)	0 (0.0)	0 (0.0)	88 (57.52)	159 (52.48)	67 (39.41)	155 (30.88)
Missing	0 (0.0)	0 (0.0)	0 (0.0)	0 (0.0)	0 (0.0)	0 (0.0)	5 (1.11)	5 (0.82)	2 (0.70)	0 (0.00)	9 (1.27)	15 (2.11)	59 (8.53)	67 (4.02)	112 (25.40)	664 (19.30)	3 (0.75)	0 (0.00)	0 (0.0)	0 (0.0)	0 (0.0)	0 (0.0)	234 (100.0)	468 (100.0)	0 (0.0)	0 (0.0)	0 (0.0)	0 (0.0)
**Intake of salt**																												
Low	165 (71.74)	382 (69.84)	0 (0.0)	0 (0.0)	0 (0.0)	0 (0.0)	91 (20.22)	173 (28.31)	61 (21.33)	97 (31.91)	0 (0.0)	0 (0.0)	580 (83.82)	1,106 (66.35)	88 (19.95)	988 (28.72)	176 (43.89)	244 (53.63)	153 (61.69)	359 (75.10)	102 (46.36)	218 (28.99)	71 (30.34)	158 (33.76)	76 (49.67)	190 (62.71)	68 (40.00)	222 (44.22)
Intermediate/high	65 (28.26)	163 (29.80)	0 (0.0)	0 (0.0)	0 (0.0)	0 (0.0)	357 (79.33)	437 (71.52)	221 (77.27)	202 (66.45)	0 (0.0)	0 (0.0)	84 (12.14)	146 (8.76)	241 (54.65)	1,940 (56.40)	222 (55.36)	211 (46.37)	95 (38.31)	119 (24.90)	118 (53.64)	534 (71.01)	160 (68.38)	308 (65.81)	77 (50.33)	111 (36.63)	97 (57.06)	246 (49.00)
Missing	0 (0.00)	2 (0.37)	1,016 (100.0)	1,159 (100.0)	110 (100.0)	100 (100.0)	2 (0.44)	1 (0.16)	4 (1.40)	5 (1.64)	711 (100.0)	711 (100.0)	28 (4.05)	415 (24.90)	112 (25.40)	512 (14.88)	3 (0.75)	0 (0.00)	0 (0.0)	0 (0.0)	0 (0.0)	0 (0.0)	3 (1.28)	2 (0.43)	0 (0.00)	2 (0.66)	5 (2.94)	34 (6.77)
**Dietary intake of vitamin C (mg/day), median (IQR)**	120.4 (74.2)	124.6 (87.5)	64.1 (49.5)	74.3 (53.0)	198.4 (168.6)	224.5 (100.0)	122.2 (62.6)	123.0 (63.7)	52.7 (35.5)	82.3 (65.4)	22.6 (22.9)	21.5 (21.7)	95.9 (61.2)	115.1 (72.1)	145.6 (113.5)	150.3 (114.3)	80.5 (52.5)	92.5 (50.7)	190.7 (113.1)	153.7 (119.7)	289.3 (201.4)	279.4 (205.5)	213.9 (187.6)	219.3 (183.8)	118.2 (132.2)	134.0 (105.1)	109.6 (94.6)	109.1 (89.3)
**Dietary intake of vitamin C (mg/day), quartile**																												
Quartile 1	57 (24.8)	137 (25.1)	335 (33.0)	290 (25.0)	47 (42.7)	25 (25.0)	109 (24.2)	153 (25.0)	153 (53.5)	76 (25.0)	171 (24.1)	177 (24.9)	259 (37.4)	400 (24.0)	94 (21.3)	760 (22.1)	136 (33.9)	114 (25.1)	28 (11.3)	120 (25.1)	39 (17.7)	188 (25.0)	71 (30.3)	117 (25.0)	52 (34.0)	62 (20.5)	45 (26.5)	126 (25.1)
Quartile 2	63 (27.4)	137 (25.1)	272 (26.8)	292 (25.2)	14 (12.7)	25 (25.0)	118 (26.2)	153 (25.0)	75 (26.2)	76 (25.0)	168 (23.6)	176 (24.8)	155 (22.4)	400 (24.0)	81 (18.4)	760 (22.1)	114 (28.4)	114 (25.1)	55 (22.2)	119 (24.9)	65 (29.6)	188 (25.0)	50 (21.4)	117 (25.0)	32 (20.9)	82 (27.1)	39 (22.9)	125 (24.9)
Quartile 3	63 (27.4)	137 (25.1)	208 (20.5)	289 (24.9)	16 (14.6)	25 (25.0)	111 (24.7)	153 (25.0)	32 (11.2)	76 (25.0)	173 (24.3)	177 (24.9)	144 (20.8)	400 (24.0)	85 (19.3)	760 (22.1)	62 (15.5)	114 (25.1)	77 (31.1)	120 (25.1)	49 (22.3)	188 (25.0)	60 (25.6)	117 (25.0)	28 (18.3)	86 (28.4)	44 (25.9)	126 (25.1)
Quartile 4	47 (20.4)	136 (24.9)	201 (19.8)	288 (24.9)	33 (30.0)	25 (25.0)	112 (24.9)	152 (24.9)	24 (8.4)	76 (25.0)	195 (27.4)	176 (24.8)	75 (10.8)	400 (24.0)	79 (17.9)	760 (22.1)	86 (21.5)	113 (24.8)	88 (35.5)	119 (24.9)	67 (30.5)	188 (25.0)	53 (22.7)	117 (25.0)	41 (26.8)	73 (24.1)	42 (24.7)	125 (24.9)
Missing	0 (0.0)	0 (0.0)	0 (0.0)	0 (0.0)	0 (0.0)	0 (0.0)	0 (0.0)	0 (0.0)	2 (0.7)	0 (0.0)	4 (0.6)	5 (0.7)	59 (8.5)	67 (4.0)	102 (23.1)	400 (11.6)	3 (0.8)	0 (0.0)	0 (0.0)	0 (0.0)	0 (0.0)	0 (0.0)	0 (0.0)	0 (0.0)	0 (0.0)	0 (0.0)	0 (0.0)	0 (0.0)

IQR: interquartile range

Supplementary Table 3. Results of the interaction analysis between dietary intake of vitamin C and other selected factors.

Stratum	Intake of vitamin C<br>Higher than median<br>OR (95% CI)	 Lower than median<br>OR (95% CI)	RERI (95% CI)	AP (95% CI)
**Age**			-0.01 (-0.19, 0.16)	-0.01 (-0.14, 0.12)
≤60 years	1.00 (ref)	1.13 (0.99, 1.29)		
>60 years	1.25 (1.12, 1.40)	1.37 (1.20, 1.55)		
**Sex**			0.07 (-0.09, 0.24)	0.05 (-0.07, 0.18)
Female	1.00 (ref)	1.07 (0.94, 1.22)		
Male	1.17 (1.03, 1.32)	1.31 (1.15, 1.49)		
**Socioeconomic status**			0.22 (-0.03, 0.47)	0.11 (-0.02, 0.24)
High	1.00 (ref)	1.04 (0.83, 1.29)		
Low/intermediate	1.76 (1.50, 2.06)	2.02 (1.70, 2.39)		
**Tobacco smoking status**			-0.10 (-0.28, 0.07)	-0.10 (-0.27, 0.07)
Non-current	1.00 (ref)	1.13 (1.01, 1.26)		
Current	1.01 (0.89, 1.15)	1.04 (0.91, 1.19)		
**Alcohol drinking status**			0.02 (-0.16, 0.19)	0.01 (-0.12, 0.15)
Never	1.00 (ref)	1.11 (0.96, 1.28)		
Ever	1.12 (0.99, 1.28)	1.25 (1.08, 1.43)		
**BMI**			0.35 (0.12, 0.58)	0.19 (0.08, 0.31)
Overweight/obese	1.00 (ref)	1.10 (0.97, 1.24)		
Normal weight	1.35 (1.19, 1.53)	1.80 (1.56, 2.06)		
***H. pylori* seropositivity**			0.14 (-0.19, 0.46)	0.11 (-0.15, 0.36)
Negative	1.00 (ref)	1.10 (0.82, 1.48)		

Positive	1.08 (0.86, 1.37)	1.32 (1.02, 1.71)		
**Intake of fruit and vegetables**			0.13 (-0.10, 0.37)	0.09 (-0.07, 0.25)
High	1.00 (ref)	1.15 (0.96, 1.36)		
Intermediate/low	1.22 (1.09, 1.37)	1.50 (1.36, 1.65)		
**Intake of salt**			0.16 (-0.17, 0.49)	0.09 (-0.10, 0.29)
Low	1.00 (ref)	1.28 (1.12, 1.47)		
Intermediate/high	1.24 (1.03, 1.50)	1.68 (1.39, 2.03)		

RERI: relative excess risk due to interaction, AP: attributable proportion due to interaction, OR: odds ratio, CI: confidence interval, BMI: body mass index

Supplementary Figure 1. Flowchart describing inclusion of studies and participants.

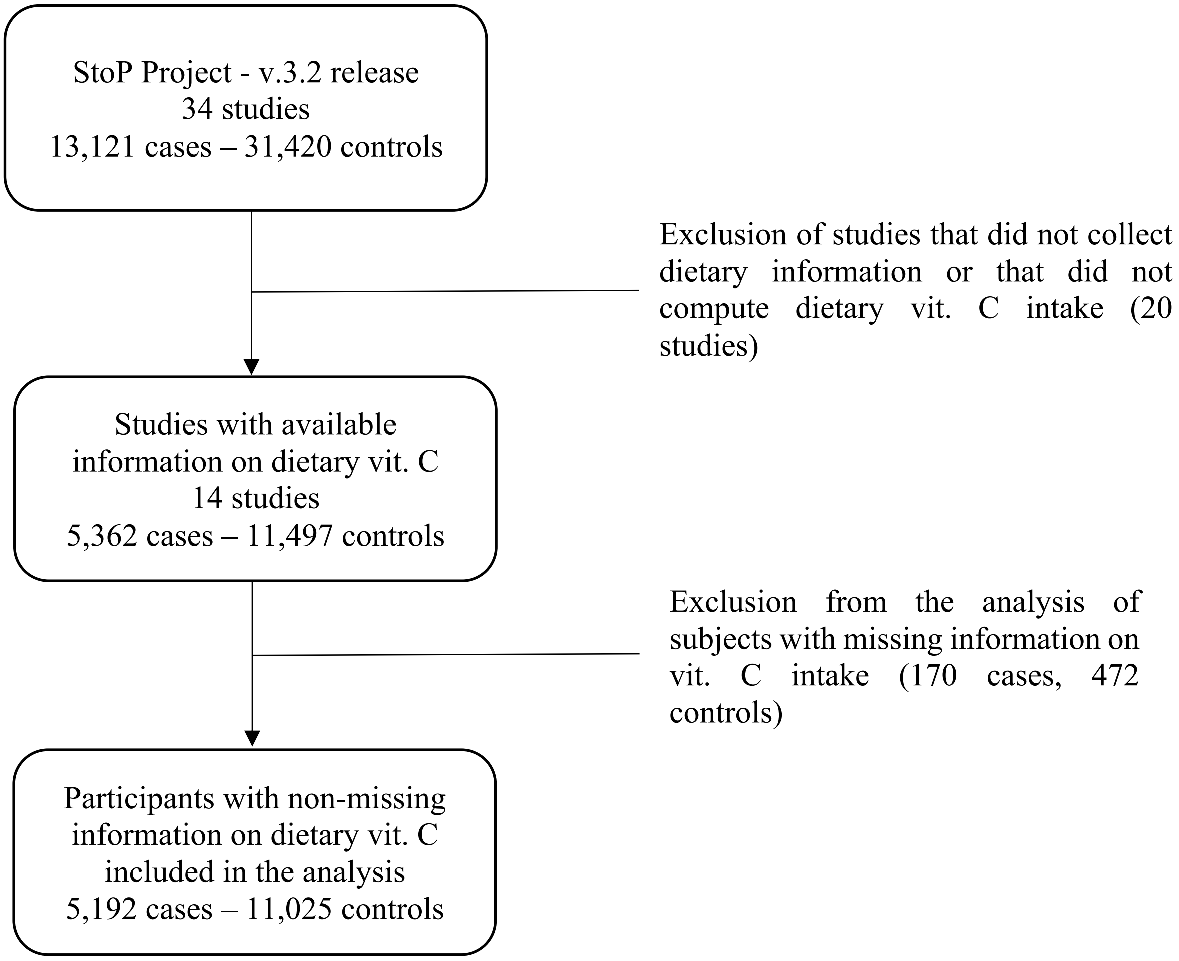

Supplementary Figure 2. E-value for the point estimate and the upper limit of the 95% confidence interval for the observed association between dietary intake of vitamin C and gastric cancer.

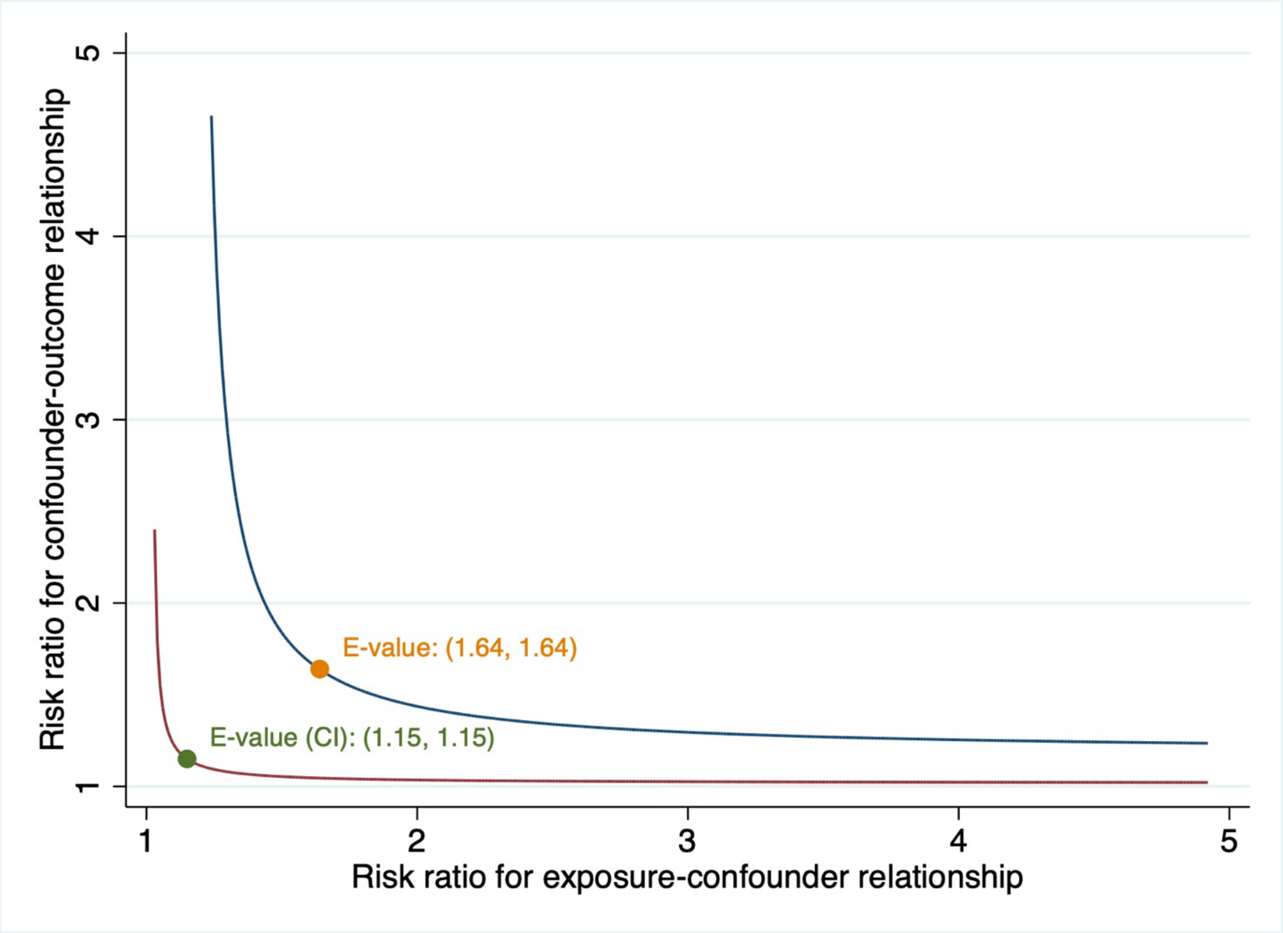

Computation of the E-value was based on estimates from Model 2 (adjusted for sex [male, female], age [≤40 years, 41-50 years, 51-60 years, 61-70 years, >70 years, missing], socioeconomic status [low, intermediate, high, missing], tobacco smoking status [never, former, current, missing], alcohol drinking status [never, ever, missing], body mass index [underweight, normal weight, overweight, obese, missing], intake of fruit and vegetables [low, intermediate, high, missing], total energy intake [continuous]).
